# Machine learning and Voronoi-based decision boundaries for Bacterial vaginosis to determine population- specific microbial interactions

**DOI:** 10.1371/journal.pcbi.1014767

**Published:** 2026-10-05

**Authors:** Cameron G. Celeste, Carleigh C. Sokolik, Wambui Gachunga, Ivana K. Parker

**Affiliations:** 1 J. Crayton Pruitt Department of Biomedical Engineering, University of Florida, Gainesville, Florida, United States of America; 2 Emerging Pathogens Institute, University of Florida, Gainesville, Florida, United States of America; 3 Department of African American Studies, University of Florida, Gainesville, Florida, United States of America; University of Nebraska-Lincoln, UNITED STATES OF AMERICA

## Abstract

In this study we utilize machine learning techniques to create predictive models and determine key bacterial interactions for the diagnosis of Bacterial vaginosis. Bacterial vaginosis (BV) is a common vaginal syndrome affecting reproductive-age women globally. It is associated with various adverse obstetric and gynecological out-comes including increased risk of sexually transmitted infections, HIV, cervical cancer, and pre-term birth. While it is known that BV is caused by a shift in abundance between Lactobacilli and anaerobic bacteria, it is unknown how gradual shifts in that balance lead towards BV status. Here we perform a rigorous comparison of machine learning architectures and feature selection methods used to train models on 16s rRNA data of patients presenting with BV. Using the highest-performing models, we employ explainable AI methods to determine the most important bacteria for BV diagnosis. Furthermore, we implement Voronoi-based decision boundaries to show how the relative abundances between pairs of these bacteria results in BV positive or BV negative outcomes. Results: We find that support vector machine and random forest models in combination with feature selection predict BV diagnosis with the most balanced accuracy. Using those models, we identify four Lactobacilli spp and six anaerobes to be key in to be key to the diagnosis of BV. The determination of key bacteria can inform BV diagnostics and pathogenesis research to species that have previously eluded scientific focus. Additionally, decision boundary plots offer a diagnostic point of reference for how the relative abundances of key vaginal flora are indicative of BV outcomes.

## Author summary

Bacterial vaginosis (BV) is one of the most common vaginal syndromes among women of reproductive age. It impacts 23% to 29% of women globally and is linked to an increased risk of gynecological complications. Over the past decades research has yielded innovations in BV diagnosis and treatment, yet very little is known about how the microbial species interact with each other to progress into BV. Additionally, while evidence suggests that women have complex and diverse vaginal microbiomes that can vary in a population specific manner, many diagnostic methods and treatments ignore personalized factors. Here, we use machine learning models across populations to identify important microbes for BV diagnosis using machine learning. Furthermore, we characterize how abundances of these microbes determines BV. To do this, we identify the best models for each population among the 150 trained to determine BV in patients. We use these models to identify the bacteria that are most important in BV using feature importance. We highlight 10 bacteria that are important for BV determination, 3 of which have not previously been studied in the context of BV. We also use a novel method to create machine learning decision boundary plots to show how the abundance of bacterial pairings can be indicative of BV. With further accompanying research, these plots have the potential to be used as a diagnostic tool or screening reference.

## Introduction

Bacterial vaginosis (BV), one of the most common vaginal syndromes worldwide, is characterized by an imbalance in the vaginal microbiota, affecting an estimated 23% to 29% of women of reproductive age globally [1]. BV presents with a decrease in Lactobacilli and an overgrowth of anaerobic bacteria posing significant implications for women’s health, including broader gynecological and obstetric risks [2–5]. Women with BV face an increased likelihood of contracting sexually transmitted infections, including HIV [6,7] and adverse pregnancy outcomes such as preterm birth. Despite these associations, the pathogenesis of BV is inadequately understood.

Diagnostics for BV have historically relied on Amsel’s Criteria [8] and the Nugent Scoring System [9]. Amsel’s Criteria evaluates clinical symptoms such as vaginal discharge, pH, and microscopic indicators like clue cells, whereas Nugent Scoring grades bacterial morphotypes in Gram-stained vaginal smears to assess microbial composition. While these methods provide the foundation for BV diagnostics, they are not without limitations. The vaginal microbiome is extremely complex, shown to vary by race and ethnicity, with women of African descent having more complex vaginal microbiomes in a healthy state [10]. As both Nugent scoring and Amsel’s criteria were validated using predominantly European cohorts [8,9], there is an opportunity to characterize healthy bacterial communities in diverse groups of women to ensure accurate diagnosis.

Asymptomatic BV is characterized by a diagnosis based on Nugent scoring in the absence of clinical symptoms defined by Amsel’s criteria, such as abnormal vaginal discharge or malodor [11]. A 2001–2004 survey of BV prevalence in the U.S. found that of 21 million women with BV, 84.3% of them were asymptomatic [12]. The high prevalence of asymptomatic BV raises important questions regarding the definition of a “healthy” vaginal microbiome, particularly in the context of clinical management and treatment decision-making [13]. Moreover, given documented variation in vaginal microbiome composition across populations, it remains unclear whether definitions of BV based on small subsets of women (predominantly European) are universally appropriate. This further highlights the need for improved diagnostic frameworks and a more nuanced understanding of BV pathogenesis and progression across diverse populations.

Advances in molecular biology have transformed the diagnostic approaches for BV. Techniques such as 16S rRNA sequencing and quantitative PCR (qPCR) [14,15] allow for the identification of bacterial species with high precision and provide detailed insights into the dynamics of the vaginal microbiota. These tools have laid the groundwork for integrating computational approaches, particularly artificial intelligence, into BV research. Machine learning models have demonstrated potential in analyzing high-dimensional microbiome data and identifying predictive patterns of BV-associated bacterial imbalances [16,17]. Recent work has further emphasized the utility of explainable AI methods in addressing ethnic disparities in BV diagnosis. Celeste et al. found that although ML models show potential for predicting BV, their effectiveness varies significantly across ethnic groups [18]. These differences in ML model performance show a need to explore how different bacterial populations impact BV diagnosis across ethnic groups.

Explainable AI offers a way to further explore these differences and determine which bacterial species are important to BV diagnosis. The goal of explainable AI is to better interpret the logic behind machine learning model decisions [19]. When interpreting how models behave, there are a number of model agnostic techniques that can explain the decision making process of any model [20]. In this paper, we will utilize permutation importance. This method permutes the variable vectors to estimate their individual importance on the model outputs [21]. Here, using permutation importance, we are able to identify the various bacterial species that impact ML model performances across each population.

Furthermore, it is possible to visualize the way ML models utilize the relationship between these species by plotting their decision boundaries. For lower dimensional models, such as those using only two or three variables to make predictions, the decision boundary is a line or a plane that shows how these variables are used to separate each sample into one classification or the other. Higher dimensional models use hyperplanes to describe those decision boundaries, which are harder to visualize and not intuitive to the viewer. Traditionally, in order to visualize these boundaries, dimensional reduction methods such as t-sne, PCA, and UMAP have been used to cluster variables into ambiguous components [22–24]. Unfortunately, with these methods, individual variables are not able to be observed independently of their clusters. To visualize how individual bacteria interact to determine BV, we utilize a method developed by Migut et al [25]. This method uses Voronoi tessellation to create two dimensional decision boundaries from higher dimension models. These Voronoi-based decision boundaries do not cluster variables, and therefore individual bacterial relationships are able to be visualized.

In this study, we utilize machine learning models to characterize key bacterial relationships in determining BV outcomes for patients across multiple ethnic populations. We do this by training machine learning models to classify BV outcomes based on 16s rRNA sequencing data. Multiple machine learning models are trained to identify the best combination of model architecture and feature selection method for each population based group. Once the best models are determined, we utilize permutation importance to identify key bacteria for each group. Voronoi-based decision boundaries are used to determine how the machine learning models are using these bacterial interactions to determine BV diagnosis.

In total, 150 machine-learning models were trained and evaluated using n = 10 repeats for each model. Through these models, 10 bacteria were identified as most important for determining BV. From these bacteria, a total of 28 Voronoi-based decision boundary plots were created. By measuring the relative abundance of a few vaginal microbes, these Voronoi-based decision boundary plots can be utilized to determine BV diagnosis in patients. Furthermore, future studies of these key bacteria and Voronoi-based decision boundaries can lead to molecular diagnostic tools for BV that are accurate for women of varying populations.

## Results

### Large scale machine learning determines highest performing models

In this study we utilize machine learning to identify key bacterial relationships in the diagnosis of BV. We rigorously train and evaluate high-performing machine learning classifiers utilizing feature selection and observe which bacteria are treated as most important. In order to reduce machine learning biases and to observe potential population-specific differences, we trained 150 models that cover a wide scope of ML algorithms and pre-training methods to classify BV diagnosis on population specific subsets. All of the models were trained using 16s rRNA derived relative abundance data from two previously published datasets [14,15]. Each sample represents a vaginal swab taken from a woman with or without BV. Each feature represents the relative abundance of a bacterial taxa within the vaginal microbiome. The two datasets were merged by aligning features at the lowest shared taxonomic level, producing a combined set of 616 vaginal microbiome samples with 67 features, largely at the genus level. Some taxa (e.g., Actinobacteria, Clostridia) were grouped at higher taxonomic ranks. While this method for combining features reduced taxonomic resolution, it enabled inclusion of key contributors towards BV from both datasets.

Using the combined dataset, we trained and assessed the performance of five different machine learning architectures: Logistic regression (LR), random forest (RF), support vector machine (SVM), multilayer perceptron (MLP), and XGBoost (XGB). We also utilize five different statistical feature selection methods in order to improve the performance of these models. These methods include ANOVA F-test (Ftest), two-sided T-Test (Ttest), Point Biserial correlation (Pbcorr), Point Biserial significance (Pbsig), and the Gini impurity (Gini). These statistical feature selection tests used in this study were chosen to provide transparent reduction of high-dimensional data prior to model training. These methods evaluate each feature independently and therefore do not explicitly model feature–feature interactions. However, the classifiers employed are capable of capturing interactions among features during training. Regression based methods such as LASSO conflate feature selection with a specific model architecture and optimization objective [26], which would limit cross-model comparability and interpretability across populations. In order to prioritize explainability, statistically interpretable feature selection methods were prioritized. The five population specific subsets are identified as “White”, “Black”, “Asian”, and “Other”, with the set titled “Total” representing the overall dataset. For each combination of population, model architecture, and feature selection method, 10 replicates were trained for statistical analysis. In total 1500 models were trained, optimized, and tested, to evaluate the 150 possible combinations. Tukey box plots of the balanced accuracy for these models are shown in Fig 1. The exhaustive list of model performances for all 1500 models is accessible in S1 Table.

**Fig 1:**
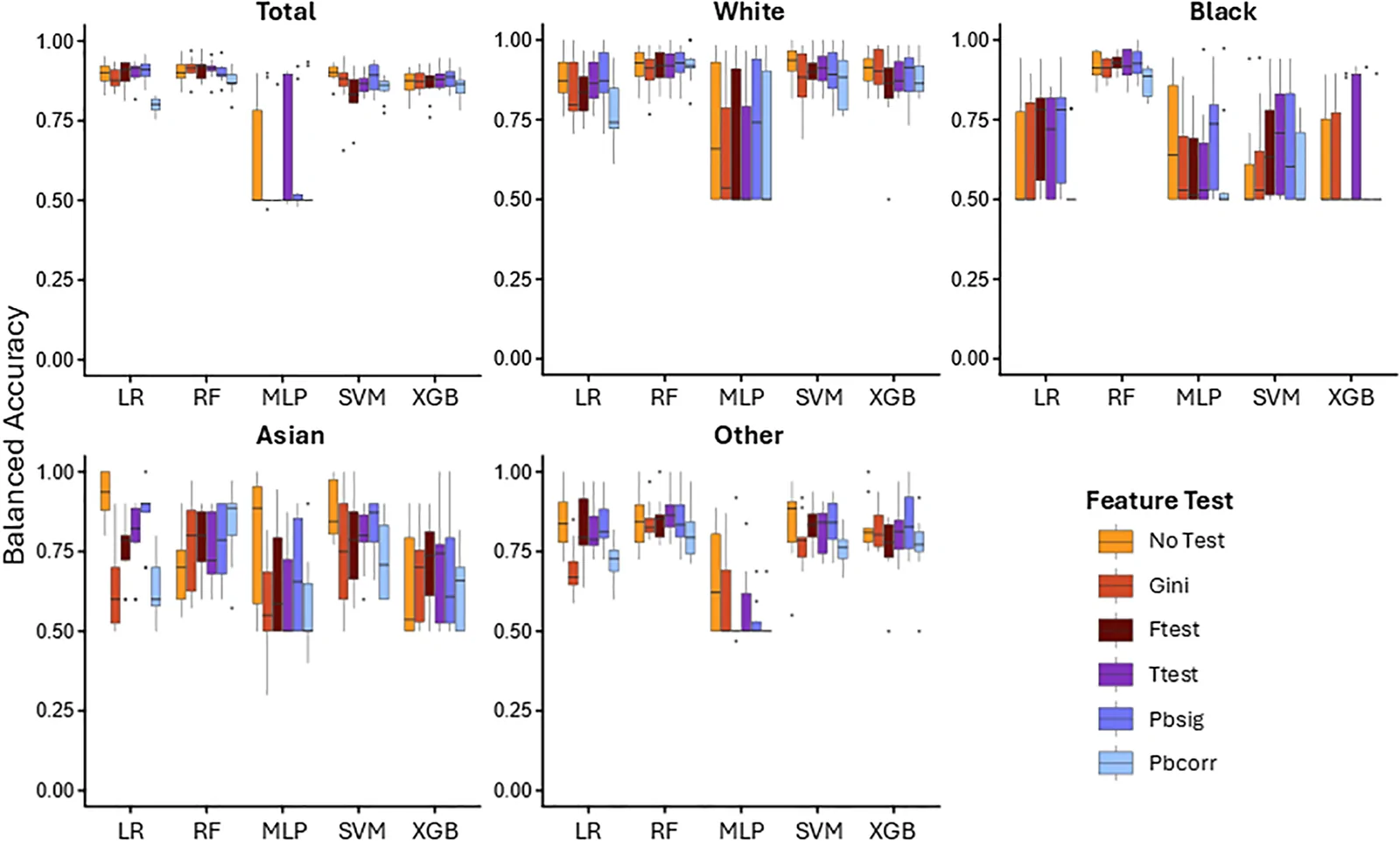
Performance for all ML Models classifying patients as BV positive or negative. Models were trained and tested against each group with n = 10 repeats. Boxplots for the Total, White, Black, Asian, and Other population groups show ML algorithm on x-axis and balanced accuracy on y-axis. Boxplots are grouped and colored by pre-training feature test method. The whiskers denote 1.5x IQR above the 75^th^ quartile and below the 25^th^ quartile. If this value is below the minimum or above the maximum, the whiskers represent those values instead.

For later permutation importance evaluation, we identify the best combination of model architecture and feature selection methods for each group. The best model architectures overall were the RF, with LR performing better only for the Asian group. For the Total dataset, the RF model with Gini feature selection yielded the highest performance (BA 0.914). For the Other group, the RF model with Ttest feature selection yielded the highest performance (BA 0.864). For the Asian group, an LR model using no feature selection (BA 0.932) yielded the best results. For the White group and Black group models, RF with Pbsig feature selection yielded the best model (BA 0.923 and 0.930). Tukey box plots and balanced accuracy for the highest-performing models are shown in Fig 2 and Table 1.

**Table 1:** Balanced accuracy of the best machine learning models. The model architecture and statistical feature test are listed.

Population	Feature Test	Model	Balanced Accuracy
Total	Gini	RF	0.9142
White	Pbsig	RF	0.9228
Black	Pbsig	RF	0.9303
Asian	No Test	LR	0.9317
Other	Ttest	RF	0.8638

**Fig 2:**
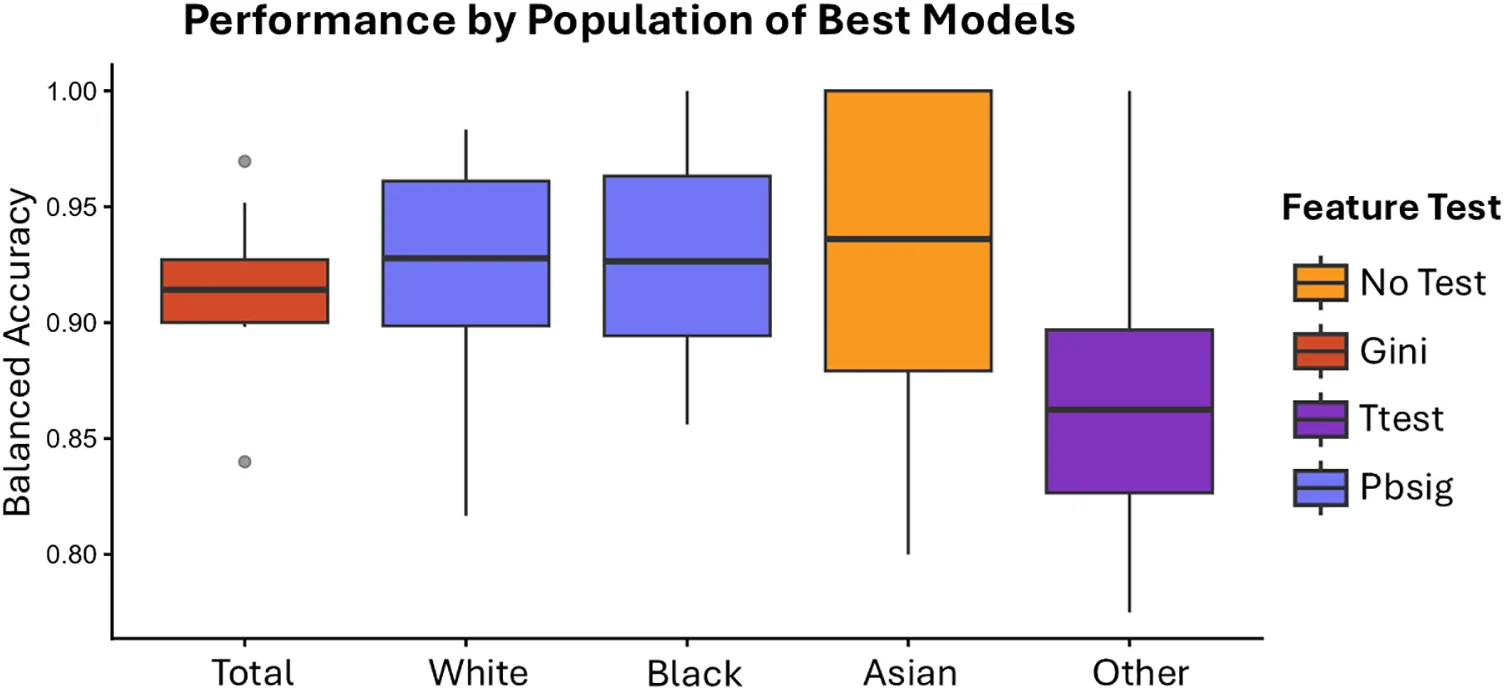
Balanced Accuracy boxplots and corresponding combinations of the best-performing models. Models were trained and tested against each group with n = 10 repeats. Boxplots are colored by the feature selection test used to select the x labels. The whiskers denote 1.5x IQR above the 75^th^ quartile and below the 25^th^ quartile. If this value is below the minimum or above the maximum, the whiskers represent those values instead.

Fig 2 shows that across populations, the highest performing model configurations also demonstrated relatively consistent performance distributions across repeats, with limited spread in balanced accuracy.

### Permutation importance reveals key bacteria for BV prediction in each population

The best models for each group were then analyzed for feature importance. Feature importance reveals how these machine-learning models leverage each feature for prediction. In this study, more key features correspond to bacteria that are key to the determination of BV in each group. We utilize a method of feature importance known as permutation importance, where values in each feature space are shuffled randomly before models are tested. An increased degradation from shuffling indicates increased importance of a feature to the machine learning model. Permutation importance scores for the highest performing models are shown in Table 2.

**Table 2:** Key Bacteria Determined using Permutation Importance. The table shows the permutation importance score of bacteria for each group. The score represents the reduction in balanced accuracy due to shuffling of the values corresponding to that bacteria. A blank means the bacteria did not score as important for that group.

	Population Group				
Bacteria	Total	White	Black	Asian	Other
*Eggerthella (Combined)*	0.031972	0.043765	0.035960	---	0.026875
*Gardnerella (Combined)*	0.061990	0.084722	0.057740	---	0.033063
*Lactobacillales (Combined)*	0.010048	---	0.020566	---	0.050000
*Lactobacillus (Combined)*	0.006777	---	---	---	---
*Lactobacillus crispatus*	0.007682	0.021852	0.013061	0.104074	0.034821
*Lactobacillus iners*	0.005850	---	---	---	---
*Megasphaera*	0.010189	---	---	---	---
*Parvimonas (Combined)*	0.011582	0.019630	0.015546	---	0.013854
*Lactobacillus jensenii*	0.005645	---	---	0.044691	---
*Prevotella*	0.003304	---	---	---	---
*Mycoplasmataceae*	---	0.018241	---	---	---
*Gemella (Combined)*	---	---	0.012207	---	---
*Lactobacillus gasseri*	---	---	0.014886	0.143611	---
*Lactobacillus vaginalis*	---	---	0.012119	0.204667	0.036875
*Leptotrichia (Combined)*	---	---	---	0.020079	---
*Streptococcus*	---	---	---	0.024236	---

Permutation importance reveals 16 features as key to the diagnosis of BV. The Total dataset group had the most bacteria considered important by the model. These included *Eggerthella* (Combined), *Gardnerella* (Combined), *Lactobacillales* (Combined), *Lactobacillus* (Combined), *Lactobacillus crispatus*, *Lactobacillus iners*, *Megasphaera*, *Parvimonas* (Combined), *Lactobacillus jensenii* and *Prevotella*. Of these bacteria, *Lactobacillus* (Combined), *Lactobacillus iners, Megasphaera* and *Prevotella* are uniquely important to the Total group. The White group important bacteria include *Eggerthella* (Combined), *Gardnerella* (Combined), *Lactobacillus crispatus*, *Parvimonas* (Combined) and lastly *Mycoplasmataceae*, which is unique to the White group. The important bacteria for the Black group are *Eggerthella* (Combined), *Gardnerella* (Combined), *Lactobacillales* (Combined), *Lactobacillus crispatus*, *Parvimonas* (Combined), *Gemella* (Combined), *Lactobacillus gasseri*, and *Lactobacillus vaginalis*. Of these, *Gemella* (Combined) is the only unique member of the list. For the Asian group, the important bacteria are *Lactobacillus crispatus, Lactobacillus jensenii, Lactobacillus gasseri, Lactobacillus vaginalis, Leptotrichi*a (Combined), and *Streptococcus.* These last two are unique to the Asian group. Lastly, the “Other” group, which has no unique members, includes *Eggerthella* (Combined), *Gardnerella* (Combined), *Lactobacillales* (Combined), *Lactobacillus crispatus, Parvimonas* (Combined), *Lactobacillus vaginalis*.

### Visualization of bacterial abundances driving BV prediction using Voronoi- decision boundaries

Once key bacteria were identified, decision boundaries were plotted between pairs of identified bacteria to elucidate how their relative abundances correlate to BV outcomes. Usually, to represent the decision boundary of a high-dimension model in two dimensions, it is necessary to use dimensional reduction. With dimensional reduction, however, the X and Y axes lose feature separability as they represent a combination of multiple features. This separability is necessary to understand how the relationship between individual bacteria determine BV.

Voronoi-based decision boundaries estimate the boundary between two features while maintaining separability. Fig 3 shows the Voronoi-based decision boundaries developed using the method described by Migut et al [25]. Blue zones indicate a BV-negative outcome, and red zones indicate a BV-positive outcome. The gray zone indicates regions where the combined relative abundance would total beyond 100%. Decision boundaries are grouped by Black, White, Other, and Asian groups, and then by harmful bacteria.

**Fig 3:**
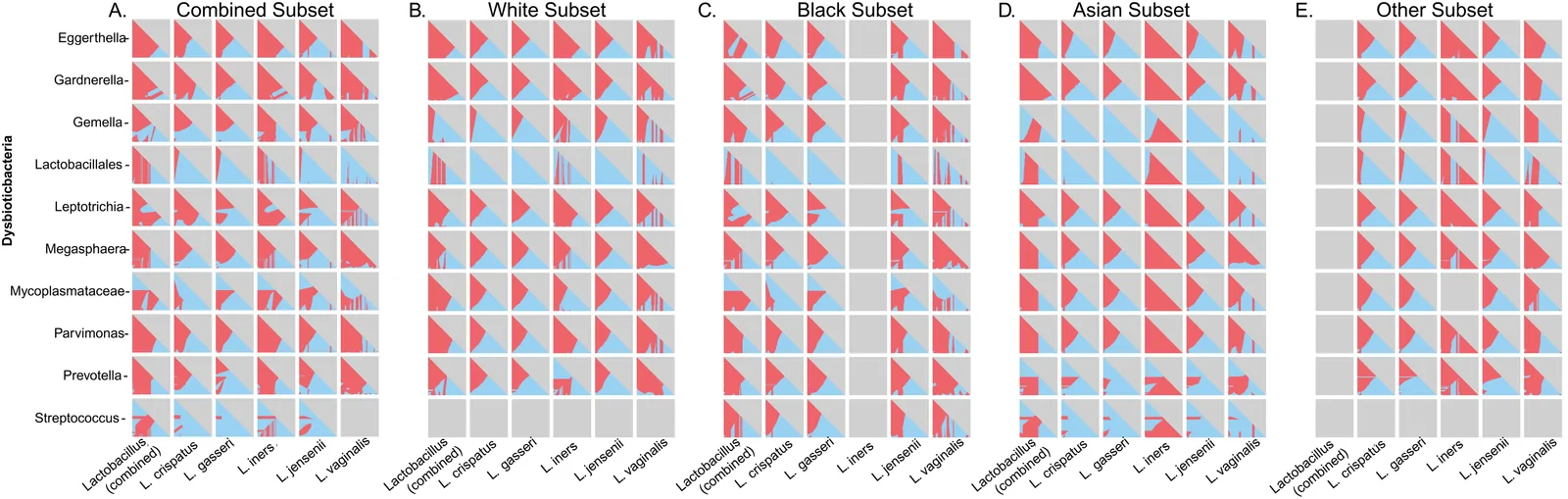
Voronoi-based decision boundaries between important bacteria for each group. Figure shows plots for Total, White, Black, Asian, and “Other” subsets (A-E). Red zones indicate a BV positive prediction by the model, while blue zones indicate a BV negative prediction. The gray zone indicates regions where the combined relative abundance would total beyond 100%. Plots that are entirely gray indicate that neither healthy nor dysbiotic bacteria were a part of the selected feature space.

Many bacterial pairings demonstrate well-defined decision boundaries, indicating that relationships between two bacteria are informative for BV discrimination. However, boundary clarity varies by microbial identity and population subset.

In the Total subset, *Mycoplasmataceae, Leptotrichia*, *Gemella*, and *Gardnerella* plots tend to exhibit poorly defined decision boundaries, reflecting reduced separability in these pairings. When *L. crispatus* is the protective lactobacilli bacteria, nearly all plots display clear boundary definition, with the exception of its pairing with *Leptotrichia*. *Lactobacillale*s demonstrates a lesser contribution towards dysbiosis, as only very high concentrations of *Lactobacillales* yield a BV positive decision. An exception is the *Lactobacillales* vs *Lactobacillus* (Combined) plot, in which even low *Lactobacillales* abundances are associated with BV. Of the dysbiotic bacteria, *Prevotella, Parvimonas*, and *Gardnerella* tend to overpower their healthy *Lactobacilli* counter parts. Low relative abundances of these bacteria are sufficient to yield BV positive decisions unless there is also a high abundance of the protective taxa.

Within the White subset, most taxa-specific plots exhibit well-defined decision boundaries, indicating robust separability. Notable exceptions include plots for *L. vaginalis*, select *Lactobacillales* relationships, and *L. iners*, where decision boundaries are less distinct. Similar to the results from the Total dataset, *Gardnerella* and *Parvimonas* exhibit a stronger influence on BV classification. In contrast, *Prevotella* contributes less to model decision-making.

In the Black subset decision panels, *Lactobacillales* follows the same trend, exhibiting poor boundary definition and minimal contribution to the classification decision. *L. vaginalis, Lactobacillus* (combined) and *Leptotrichia* plots do not have well defined boundaries, suggesting limited discriminative capacity for BV status. In contrast, the majority of remaining taxa display well-defined decision boundaries. *Gardnerella*, *Eggerthella*, and *Parvimonas* exert comparatively stronger influence on BV classification, indicating greater importance in driving model decision-making for this population.

Within the Asian subset, the majority of decision panels display well-defined decision boundaries. *L. iners* exhibits limited influence on BV classification in this subset; notably in some instances, complete (100%) abundance of *L. iners* is still associated with a BV-positive classification. Meanwhile, *L. crispatus, L. gasseri*, and *L. jensenii* exert a pronounced protective influence against BV when evaluated in pairwise comparisons against *Gemella* and *Lactobacillales*. Conversely, *Prevotella, Streptococcus*, and *L. vaginalis* plots in this subset lack clearly defined decision boundaries, suggesting weaker discriminatory capacity within this subset.

Finally, within the “Other” subset, decision panels involving *L. iners* and *L. vaginalis* plots lack clearly delineated classification boundaries. In contrast, *L. crispatus*, *L. gasseri,* and *L. jensenii* exhibit more pronounced boundary definition. *Gemella* and *Lactobacillales* demonstrate weak influence on the BV classification decision, further indicating reduced contribution to model decision making.

Collectively, these decision boundary plots illustrate how specific pairwise relationships between dysbiotic and healthy bacteria contribute to BV classification. By examining the spatial distribution of BV- positive and BV- negative regions within each panel, interactions between the two bacteria can be characterized. These patterns provide insight into how co-occurrence and relative abundance dynamics influence classification outcomes and can be leveraged to inform predictive modeling and downstream inference of BV-associated microbial states.

### Voronoi decision boundaries demonstrate cross-dataset predictive power

A validation experiment using four publicly available metagenomic datasets was implemented to assess the generalizability of the Voronoi-based decision boundary models. Fig 4 shows details on the characteristics of the validation dataset as well as the performance of the Voronoi-based decision boundary models. The dataset includes 1422 samples, 985 BV negative and 437 BV positive (4A). 347 patients are in the White population group, 915 patients are in the Black population group, 21 patients are in the Asian population group, and 52 are in the Other group (4B). The complete list of model performances is accessible in S2 Table.

**Fig 4:**
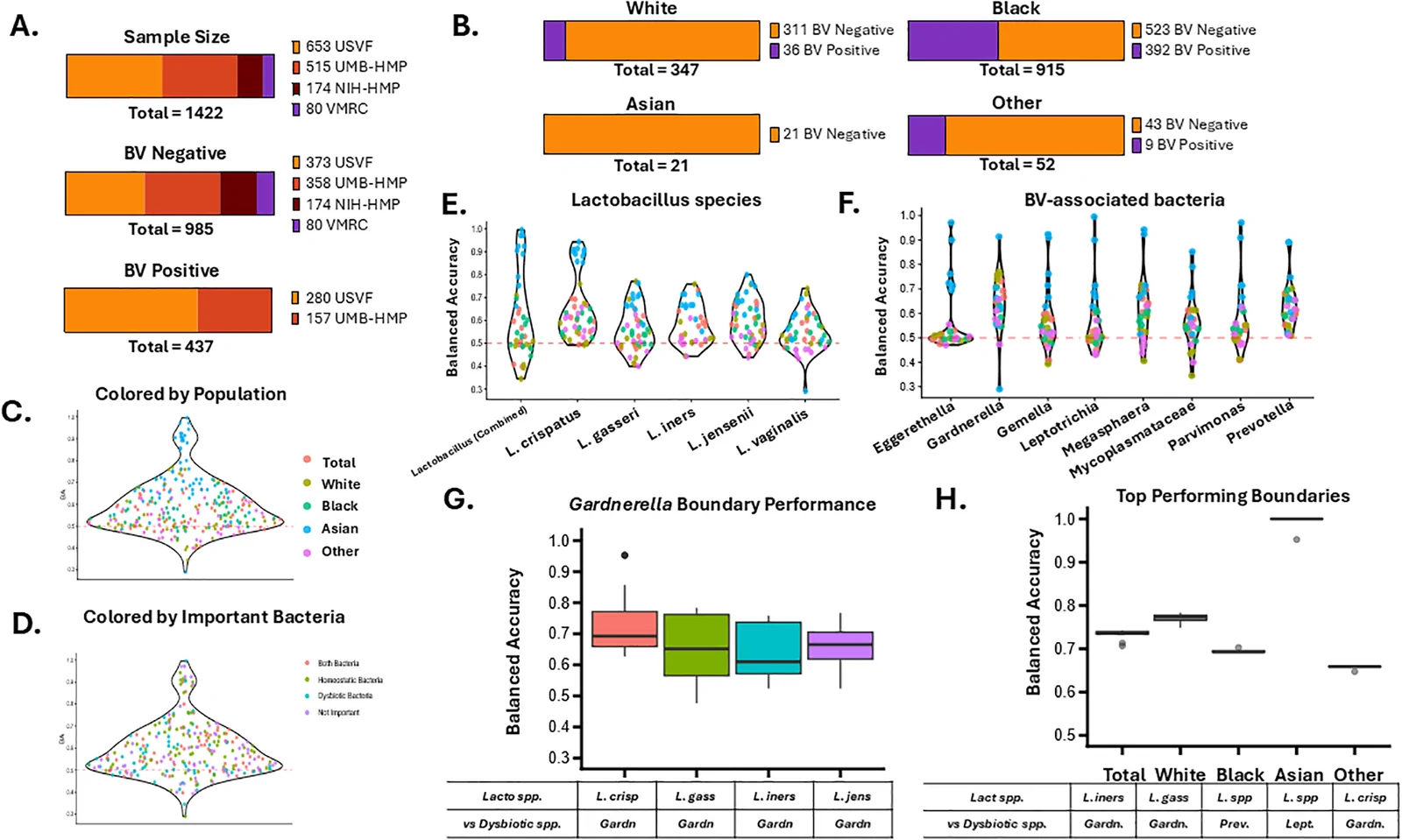
Generalizability testing of Voronoi-based decision boundaries for BV prediction. (A) and (B) show sample breakdown by dataset and population. (C-F) show violin plots for the balanced accuracy of the Voronoi-based decision boundaries in predicting BV. (C, E and F) Points of the violin plot colored are by population, while (D) shows the points of the violin plot colored by important bacteria. (E) and (F) show the same data grouped by either the healthy lactobacillus bacteria (E) or BV associated bacteria (F). Dashed red line represents balanced accuracy for random guessing. (G) shows Tukey boxplots for boundary performance using balanced accuracy for *Lactobacillus spp.* against *G. vaginalis* (H) shows the top performing boundaries for each population group.

Across the violin plots in Fig 4C and 4D, balanced accuracy distributions are characterized by a dominant density peak slightly above 0.5, with a secondary peak near 0.9. This bimodal structure suggests that there is a range in predictive performance, while a subset of decision boundary models perform near chance, others achieve substantially higher predictive performance. Among population-specific results, the Asian subset, which contains the largest percentage of BV-negative cases in the combined cohort, shows strongest overall performance. In contrast, performance across the remaining populations is more heterogeneous, with no clear or consistent trend emerging. To examine this trend, performance was also evaluated by CST in each subgroup (S1 Fig). The Asian subgroup contains higher percentages of CST I-A, I-B, II, and III-A than the other subgroups. Unlike the other subgroups, the Asian subgroup also lacks patients from CSTs III-B and IV. This imbalanced in the data within each subgroup, could contribute to each subgroup specific performance.

Fig 4D further contextualizes these results by coloring each model according to the type of bacteria (healthy, dysbiotic, or both) identified as most influential for the corresponding population-specific classifier. Across taxa, no systematic advantage is observed for any single bacterial group being designated as important, suggesting that elevated model performance is not driven solely by the presence of a particular type of organism. Instead, predictive accuracy appears to arise from more complex interactions between bacterial pairs.

Fig 4E and 4F stratify balanced accuracy distributions by healthy and dysbiotic bacteria, respectively. When grouped by healthy bacteria (Fig 4E), most taxa exhibit high density around balanced accuracies of approximately 0.5. However, models incorporating L. crispatus along the x-axis demonstrate a shift toward higher balanced accuracies, an effect that is most pronounced within the Asian subset. This pattern suggests a stronger and more informative role for *L. crispatus* based relationships in BV classification for this population.

A similar trend is observed when grouping by dysbiotic taxa (Fig 4F). Decision boundaries involving *Gardnerella, Megasphaera, Prevotella*, and *Streptococcus* display elevated accuracy densities relative to other dysbiotic organisms, indicating that these species contribute more strongly to successful BV classification across pairwise models.

Fig 4E then focuses on the models comparing the community state type defining bacteria and *Gardnerella*. These models exhibit performances within the 0.6 to 0.8 range depending on the bacteria. *L. crispatus* and *L. jensenii* developed decision boundaries show slightly higher statistical centers tighter ranges compared to *L. iners* and *L. gasseri* boundaries. Each of these boundaries however perform above the 0.5 density peak shown in Fig 4C. These results further support the importance of the comparison between these species and G. vaginalis in research and clinical settings.

Fig 4G shows the top performing bacterial pairings by population. These pairings for the Total, White, Black, Asian, and Other groups are as follows: *Gardnerella* (Combined) vs *Lactobacillus iners* (Average BA: 0.73), *Gardnerella* (Combined) vs *Lactobacillus gasseri* (Average BA: 0.77), *Prevotella* vs *Lactobacillus* (Combined) (Average BA: 0.70), *Leptotrichia* (Combined) vs *Lactobacillus* (Combined) (Average BA: 1.00), and *Gardnerella* (Combined) vs *Lactobacillus crispatus* (Average BA: 0.66). For the Total and Other groups, both bacteria in the pairing are identified as important by permutation importance, meanwhile for the White and Asian groups, only the dysbiotic bacteria is identified as important. For the Black group, neither bacteria in the pairing were identified as important. The highest performing boundary for the Black group with at least one important bacteria was *Lactobacillales* (Combined) vs *Lactobacillus jensenii* (Average BA: 0.68).

Collectively, these results indicate that Voronoi-based decision boundary models can capture clinically relevant signals from metagenomic abundance data, with predictive performance dependent on both species selection and population subset. While overall accuracy varies substantially across models, the presence of higher performing boundaries supports the broader generalizability of this approach. Importantly, these findings demonstrate that decision boundaries derived from one cohort retain insights when applied to samples generated using shotgun metagenomic sequencing. Together, these results highlight the potential of decision boundary visualization as a transferable and interpretable framework for BV classification across diverse populations and data sources.

## Discussion

In this study, we trained and evaluated 150 machine learning models to classify and characterize bacterial vaginosis (BV) across multiple populations using 16S rRNA derived relative abundance profiles. Permutation importance was subsequently used to identify 16 bacterial species that contributed to BV classification across high‑performing models. Utilizing Voronoi-tessellation, we visualized how relative abundance relationships between these bacteria influence BV classification. Finally, we demonstrate that these Voronoi-based decision boundaries retain predictive utility when applied to metagenomic datasets.

### Key bacteria associated with BV and related clinical contexts

Permutation importance on the highest performing machine learning models identified 16 bacterial taxa that are key to the diagnosis of BV. These bacteria include the four *Lactobacilli spp* that have dominance over vaginal microbiomes traditionally considered healthy. *L. crispatus, L. gasseri, L. iners*, and *L. jensenii* are the dominant species corresponding to community state types I, II, III, and V respectively as defined by Ravel et al [15]. Additional bacteria identified here have been previously associated with BV including *G. vaginalis, and Prevotella* [14,27,28]. Among these, *G. vaginalis* is the most frequently associated with BV, and has been extensively studied as the primary vehicle of pathogenesis [29–31]. This notion however has been challenged by the presence of *G. vaginalis* in healthy patients, especially those of African descent [10,32]. Emerging studies propose a synergy between *Gardnerella spp* and other anaerobes, particularly *Prevotella spp* [33].

Several prior studies have sought to identify bacterial features most strongly associated with machine‑learning‑based BV classification across established datasets. Celeste et al utilizes statistical t-test feature selection in order to identify the highest ranking 50 bacterial species of statistical significance to Nugent scoring classification in the Ravel set [18]. Ojo et al utilizes gini index to identify these features for the Srinivasan dataset [34]. Beck and Foster use Pearson correlation to identify the highest ranking 15 features for both the Ravel set and the Srinivasan set. The Beck and Foster analysis includes non-bacterial features such as pH and BV related symptoms [17]. These lists each highlight *Lactobacilli spp*, and *G. vaginalis. Prevotella* also appears in multiple analyses however with reduced certainty. In this study, we also identify *Gamella*, and *Mycoplasmatacae* as important bacteria*.*

### Insights from paired bacteria decision boundaries and screening potential

The Voronoi-based decision boundaries demonstrated that BV risk can in part be interpreted through pairwise relationships between healthy and dysbiotic taxa while preserving feature separability. Across all groups, *Gardnerella* and *Parvimonas* consistently showed strong associations with BV positivity, even when present at low relative abundances. These bacteria frequently dominated their healthy counterparts, which suggests that their capacity to disrupt the vaginal microbiome operates at relatively low thresholds. *Gardnerella* is known to be a important pathogenic microbe in BV etiology, however, *Parvimonas* is less studied. Among *Parvimonas* species, *Parvimonas micra (P. micra)* is an anaerobic bacterium typically found in the mouth and gut microbiomes, but it can also appear in the vaginal microbiome [35]. While not considered a primary BV organism, *P. micra* can participate in polymicrobial biofilms, which may indirectly contribute to vaginal dysbiosis [36]. Its reliance on neighboring microbes for biofilm integration suggests a secondary but potentially amplifying role in BV‑associated infection dynamics. *Prevotella* also showed strong influence in the overall dataset, although this effect was reduced in the White subset. Emerging studies propose a synergy between *Gardnerella spp* and other anaerobes, particularly *Prevotella spp* [33].

The protective roles of *Lactobacillus* species varied by population. *L. crispatus, L. gasseri,* and *L. jensenii* produced clear boundaries in most groups and effectively expanded BV negative regions across many bacterial pairings. These species appear to function as reliable indicators of vaginal homeostasis in across multiple population groups. In contrast, *L. iners* showed substantial instability. In the Asian subset, even high abundance of *L. iners* corresponded to BV positive outcomes, which aligns with previous evidence suggesting that *L. iners* provides weaker protection and may facilitate transitions toward dysbiosis.

Subgroup specific patterns further highlight the importance of differences in model interpretation between groups. In the White and Black subsets, *Gardnerella* and *Parvimonas* maintained strong predictive roles, while *Eggerthella* showed additional influence in the Black subgroup. *Eggerthella* species are highly prevalent in bacterial vaginosis, appearing in approximately 85–95 percent of BV cases [37]. They produce biogenic amines such as putrescine and cadaverine that elevate vaginal pH and contribute to the characteristic odor of BV. Experimental studies demonstrate that *Eggerthella* increases inflammatory mediators including IL1α in cervical epithelial models, indicating an active role in BV associated inflammation. Together, these known metabolic and immunologic disruptions could be important to study in a population specific manner to better understand BV dysbiosis [37].

Several taxa, including *L. vaginalis, L. iners*, and *Leptotrichi*a, frequently produced poorly defined boundaries in specific groups. These inconsistencies likely reflect a combination of underlying biological and compositional variation, and differences introduced during feature selection within each subset. *Leptotrichia* and related *Leptothrix* species occur in the vaginal environment but are relatively uncommon and often associated with non-dysbiotic states rather than BV. Studies show that *Leptothrix* is associated with a reduced risk of BV and an increased susceptibility to candidiasis, suggesting it behaves differently from classical BV associated anaerobes [38]. Other species such as *Leptotrichia trevisanii* have been linked to pelvic inflammatory disease and isolated from cervical infections [39].

In the Asian and Other groups, protective lactobacilli continued to show strong and well defined boundaries, while other taxa such as *Prevotella, Streptococcus,* and *Gemella* contributed little to diagnostic separation. Notably, *Streptococcus* species are components of the normal vaginal flora and can become clinically relevant under dysbiotic states such as BV. Research indicates that *Gardnerella*, can promote upper genital tract infection by Group B *Streptococcus*, highlighting an indirect connection between BV dysbiosis and streptococcal infection risk [40]. *Streptococci* also participate in broader polymicrobial communities during BV, although they are not considered primary BV organisms [1].

*Mycoplasmataceae* produced well defined decision boundaries for the White, Asian, and Other groups. Meanwhile for the Total and Black groups these boundaries are discontinuous and poorly defined. Members of the *Mycoplasmataceae* family, such as *Mycoplasma genitalium*, frequently co-occur with BV and may benefit from the elevated vaginal pH characteristic of dysbiosis. *M. genitalium* is an emerging sexually transmitted pathogen associated with pelvic inflammatory disease, infertility, and adverse pregnancy outcomes. BV related inflammation and altered mucosal defenses may further increase susceptibility to Mycoplasma infection [41].

These findings show that BV can be characterized from interactions between specific bacterial pairs that differ across populations. They also show that models which treat taxa as universally equivalent across ethnic groups may overlook meaningful variation. The observed differences in decision boundary structure indicate that diagnostic thresholds are not uniform across populations.

Validation and generalizability studies also show the potential for the use of these decision boundaries in conjunction with relative abundance data gathered through techniques other than 16s rRNA sequencing. Although the current performance of these boundaries as predictors of BV is not sufficient for clinical diagnoses, they do provide further insights into the relationships between the bacteria involved in BV progression. These models show they can utilize the patterns from 16s rRNA derived data to predict BV in alternative data types and with fewer bacteria. This exploration of Voronoi‑based decision boundaries as classifiers is not intended to evaluate the boundaries as a clinically deployable diagnostic model, but rather to validate them as an interpretable framework for examining the persistence and transferability of BV‑associated microbial patterns across datasets and sequencing modalities, complementing more complex predictive approaches. In order to fully evaluate the ability of Voronoi-based decision boundaries to be used across unique microbiome characterizations, their development using both metagenomic and 16S rRNA sequencing data should be further explored.

These decision boundaries may serve as reference frameworks for BV screening and risk assessment. Under such a framework, patient samples could be evaluated using a small number of targeted taxa rather than requiring full microbial profiling. For example, a vaginal swab would be evaluated using qPCR to determine the relative abundance of *L. crispatus, L. iners*, and *G. vaginalis*. Once the bacteria abundances relative to each other are determined, those values can be mapped to the machine learning decision boundaries and a BV classification can be made. A diagnostic method of this kind would be easily repeatable and would remove variation due to clinical judgment. Additionally, vaginal swabbing and 16s qPCR are affordable methods that can easily be performed regularly for screening purposes. In fact there are methods that already use 16s qPCR to successfully diagnose BV [42]. Importantly, the work presented here extends beyond simple quantification by characterizing how pairwise bacterial relationships differentially influence BV outcomes across populations.

A limitation of this study is that model selection was based on mean balanced accuracy across repeated runs without explicitly incorporating variability or model stability into the selection criteria for the best performing models. Future work could explore stability aware strategies to further mitigate potential selection bias and overfitting.

## Conclusions

In this study, we applied machine learning and explainable model interpretation to identify key bacterial species and pairwise microbial relationships that distinguish BV-positive from BV-negative microbial abundances across diverse populations. We identify well-established BV-associated taxa such as *Gardnerella vaginalis* and community state type–defining *Lactobacillus* species. Additionally, our analysis highlights several under-studied anaerobes as important contributors to BV classification in population-specific models. These findings explore their potential roles in BV pathogenesis, including synergistic interactions with known BV-associated taxa and contributions to biochemical pathways that destabilize Lactobacillus-dominated states.

Importantly, the Voronoi-based decision boundary framework provides an interpretable visualization of how relative abundances between bacterial pairs influence BV classification. These decision boundaries reveal that BV status is not solely driven by the presence or absence of individual microbes, but by quantitative balance points between protective and dysbiotic species. Such observations support a model of BV as a continuum of microbial imbalance rather than a binary shift, offering a computational lens through which early stage dysbiosis may be detected.

From a diagnostic perspective, these results demonstrate the feasibility of reducing high-dimensional microbiome data into low-dimensional, biologically interpretable parings that retain predictive power across independent datasets and sequencing modalities. The ability to classify BV using relative abundances of a small number of key taxa suggests a path toward streamlined, low-cost molecular diagnostics that are less subjective than microscopy-based methods and more adaptable towards personalized medicine. By explicitly mapping bacterial abundance ratios to diagnostic outcomes, this approach complements existing BV diagnostic frameworks and provides a foundation for personalized and scalable screening tools.

## Materials and methods

### Dataset

To conduct this study, we utilized two datasets from studies by Srinivasan et al. and Ravel et al [14,15]. The Srinivasan dataset consists of 155 operational taxonomic unit variables describing the bacterial composition of the vaginal microbiotas of 220 women with and without bacterial vaginosis (BV). The Ravel dataset consists of 247 taxonomic unit variables describing the vaginal microbiotas of 396 women. In both datasets, BV was determined based on the Nugent score derived from examining Gram-stain vaginal smears. In the Srinivasan dataset, the women were recruited from the Public Health, Seattle and King County Sexually Transmitted Diseases Clinic. In the Ravel dataset, women were recruited from one of two clinical sites in Baltimore at the University of Maryland School of Medicine or one in Atlanta, at Emory University.

### Combining datasets

Dataset operational taxonomic unit variables were manipulated such that the total read contribution of each sample was maintained within a 1% difference. First, exact matches with small naming differences were identified and reserved. Higher taxonomic matches, such as that on the genus or family level, were then identified and species contributions belonging to those taxonomies were summed across samples and reserved. Variables that were not matched in the methods described were then manually grouped by their most specific shared taxonomy. A reference sheet for how these were matched can be found in the supplementary files. This method resulted in a combined set consisting of 67 taxonomic unit variables describing the vaginal microbiotas of 616 women.

### Preprocessing

Given the dataset provides the bacterial composition based on percentage between 0 and 100, the data was normalized by sample to between 0 and 1 to ensure the model learning process is not biased by the scale of the features. Nugent scores were then binarized by establishing a threshold value of 7. Nugent scores less than the threshold was identified as BV negative and was relabeled as 0. Nugent scores equal to and greater than the threshold was identified as BV positive and was relabeled as 1.

### Population specific datasets

The preprocessed dataset was then divided into four population-specific subsets. Patients were sorted into either “White”, “Black”, or “Asian” specific datasets based on ethnicity markers provided in both the Ravel and Srinivasan reports. Patients that did not fit within these three groups were sorted into an “Other” dataset. The larger all-containing dataset is also used to train models, and is referred to as the “Total” dataset. In all, a total of five datasets are created in order to train and test the models.

### Statistical pre-training feature selection

Five feature selection methods were used on the population-specific datasets before training the machine learning models. These methods include ANOVA F-test, two-sided T-Test, Point Biserial correlation, Point Biserial significance, and the Gini impurity. These tests were implemented using the statistics and scikit learn packages in Python.

#### Anova F-Test.

The ANOVA F-Test (Ftest) was used to select 15 features with the highest F-value. The formula for the Ftest is as follows:


$$\begin{array}{c} F=\frac{SSB/(k-\text{1})}{SSW/(n-k)} \end{array}$$ (1)

Where k is the number of groups, n is the total sample size, SSB is the variance between groups, and SSW is the sum of variance within each group.

#### Two-tailed T-Test.

The two-tailed T-Test (Ttest) was used to compare the relative abundances of the BV negative versus BV positive group. This test evaluates whether or not there is a statistical significance in each feature between the BV positive group and the BV negative group. The p-value is then derived from the t-value using the standard cumulative distribution function (CDF). The t-value formula is as follows and p-value formula are as follows:


$$\begin{array}{c} t=\frac{\left({\overset{―}{x}}_{\text{1}}-{\overset{―}{x}}_{\text{2}}\right)}{\sqrt{\frac{{(s_{\text{1})}}^{\text{2}}}{n_{\text{1}}}+\frac{{(s_{\text{2})}}^{\text{2}}}{n_{\text{2}}}}} \end{array}$$ (2)

$$\begin{array}{c} p=\;\text{2}\;*\;\left(\text{1}\;-\text{ CDF}\left(|t|,\text{ df}\right)\right) \end{array}$$ (3)

With ${\overset{―}{\text{x}}}_{\text{1},\text{2}}$ being the mean, ${\text{s}}_{\text{1},\text{2}}$ as the standard deviation, and ${\text{n}}_{\text{1},\text{2}}$ being the number of samples in the two groups. In the p-value equation, df is the sum of n_1_ and n_2_ minus 2. Any feature p < 0.05 is selected for training and the rest are discarded.

### Point Biserial correlation and significance

The Point Biserial correlation (Pbcorr) and Point Biserial significance (Pbsig) tests are used to compare categorical against continuous data. In this case the categorical BV negative or positive classification against the continuous relative abundance data. The equation for the Point Biserial correlation coefficient, r_pb_, is as follows:


$$\begin{array}{c} r_{pb}=\frac{\left(M_{\text{1}}\;-\;M_{\text{0}}\right)}{\text{s}}\;\sqrt{pq} \end{array}$$ (4)

Where M1 and M0 are the mean of the continuous variable for the categorical variable with a value of 1 and 0 respectively. Here, s denotes standard deviation of the continuous variable, p is the proportion of samples with a value of, and q is the proportion of samples with a value of 0 to the sample set. Features with a correlation coefficient > 0.4 are selected and the rest are discarded in the Pbcorr test. For Pbsig, the correlation coefficient is first converted to a t-value using the following equation, then converted to a p-value using Equation (3) above:


$$\begin{array}{c} t=\sqrt{df}\frac{r_{pb}}{\sqrt{\text{1}-r_{pb}^{\text{2}}}} \end{array}$$ (5)

For Pbsig, features with a p-value < 0.02 are selected for training, and the rest are discarded.

### Gini impurity

Gini impurity (Gini) is a selection method that defines the impurity of the nodes in a tree classifier. To initialize the Gini test, an unoptimized decision tree classifier is trained on the training set. The Gini impurity value varies between 0 and 1 and Gini scores for each feature are derived based on the largest reduction of Gini impurity when splitting nodes. Higher reductions in the Gini value correlate to a more important feature used in prediction. The formula for Gini impurity is as follows:


$$\begin{array}{c} Gini=\text{1}-\;\sum {p_{i}}^{\text{2}} \end{array}$$ (6)

Where ${\text{p}}_{\text{i}}$ is the proportion of each class in the node. Any feature with a Gini score greater than 0 is selected for training, the rest are discarded.

### Models and training

We utilized five machine learning algorithms for predicting BV outcome: random forest (RF), logistic regression (LR), support vector machine (SVM), multilayer perceptron (MLP), and XGBoost (XGB). Training was repeated 10 times with the global random state incrementing up from 0 to 9 with each repeat. In each repeat, the chosen feature selection method is applied to the chosen dataset. Then the dataset is split using a 25% train-test split stratified by BV outcome. In order to optimize the hyperparameters, the training set was split into an optimization training and testing set using a 30% train-test split. Hyperparameters were then optimized using the Hyperopt package [43]. The balanced accuracy, area under the precision recall curve, false positive rate, and false negative rate for all model replicates tested on both the training split and the testing split are available in the supplementary files.

### Metrics

Once optimized and trained, the models make predictions on the testing set. To evaluate the performance of each model and the quality of the predictions, we used balanced accuracy (BA). BA uses both specificity and sensitivity in its calculation to score the model’s accuracy. The calculation for BA is shown in Equation 7 below. The models with the highest mean BA across repeats were identified for each population and saved using the python pickle method for later evaluation. While model performance stability is not a direct criteria for model selection, Tukey boxplots for balanced accuracy, false positive rates, and false negative rates were plotted to report model variability.


$$\begin{array}{c} Balanced\;Accuracy=\frac{\text{1}}{\text{2}}\;(\frac{True\;Positive}{True\;Positive+False\;Negative}+\frac{True\;Negative}{True\;Negative+False\;Positive}) \end{array}$$ (7)

### Statistics

The results were analyzed to determine how models using each architecture trained on different groups and using different feature selection methods differed in performance. The 6 models trained utilizing various feature selection methods for a given group and architecture will be referred to as a “cluster”. To evaluate the normality of the results, a Shapiro-Wilk test was performed on each cluster. If the test resulted in a p-value greater than or equal to 0.05, than the data for a specific model was determined to be normal. If all models in a cluster were identified as normal, then a one way ANOVA was used to determine if statistically significant differences were present in a cluster. If any of the models in a cluster did not have a normal distribution, then a Kruskal-Wallis test was used instead. If significant differences were identified, ad hoc tests were performed to determine which models in a cluster were significantly different. During ad hoc testing, if both models had normal data distributions, a one-way t-test was performed. If one or both models did not have a normal data distribution, a Wilcoxon signed-rank test was used instead. All statistical analysis of the models was performed using R.

### Permutation importance

To identify the bacteria most important in the diagnosis of BV across different groups, the models with the largest average BA for each group were evaluated using permutation importance. Permutation importance evaluates each feature one at a time by shuffling the values around the sample space. The permutation importance value is the difference between the baseline balanced accuracy and the balanced accuracy from permutating the feature column. Permutation was performed with 10 repeats, and a random state of 1. The permutation importance was implemented using sklearn’s inspection.feature_importance.

### Voronoi-based decision boundary

Voronoi-based decision boundaries are implemented to graphically represent how the machine learning models determine BV outcomes based on the relative abundance. Decision boundaries are made according to the method developed by Migut et al [25]. In this method, a K-nearest neighbor model with minimal tolerance is used to estimate the decision boundaries of high-dimension models. Training set features for the best models were used by the trained classifiers to generate a prediction set. This prediction set is then used as the ground truth label for a K-nearest neighbor (knn) classifier with k = 1. The knn classifier was given the bacteria of interest, two at a time, for the feature space. Once trained, the two-dimensional decision boundary of the knn classifier is plotted.

### Validation experiments

Voronoi-based decision boundaries were validated using a vaginal metagenomics dataset previously assembled by Ravel et al [44]. This validation cohort includes publicly available datasets from the Longitudinal Study of Vaginal Flora and Incident STI (LSVF) [45], the University of Maryland Baltimore Human Microbiome Project (UMB-HMP and VMRC), and the National Institutes of Health Human Microbiome Project (NIH-HMP) [46]. The feature space of this combined dataset includes 273 bacterial species which were grouped to match the feature space of the Ravel and Srinivasan combined dataset. The validation cohort includes 1422 samples with a total of 437 BV positive and 985 BV negative samples. The validation set was separated into population specific datasets and the decision boundary models predicted BV diagnosis only on the same population dataset. Predictions are evaluated using balanced accuracy.

## Supporting information

S1 Table Bacterial taxonomic harmonization and reference mapping. This table provides a comprehensive mapping of bacterial taxa used in the analysis, including harmonization of genus- and species-level names across reference taxonomies. Original taxonomic labels are aligned to standardized nomenclature to ensure consistency across datasets and analytical steps.
(XLSX)

S2 Table Raw model performance results across feature selection methods and population groups. This table contains the complete set of unfiltered machine-learning model outputs, including balanced accuracy (BA), average precision (AP), false positive rate (FPR), and false negative rate (FNR). Results are reported across multiple feature selection techniques, model types, random seeds, and population strata.
(CSV)

S3 Table Model performance results stratified by training–testing data splits. This table summarizes model performance metrics following repeated training–testing splits, enabling assessment of robustness and variability. Metrics are reported by feature selection method, model type, population group, and random state to support transparency and reproducibility.
(CSV)

S4 Table External validation performance across bacterial dominance boundaries. This table reports balanced accuracy (BA) from validation analyses comparing pairwise bacterial dominance boundaries (e.g., non-Lactobacillus taxa versus Lactobacillus species and combined Lactobacillus groups). Results are presented for the total sample and stratified population groups, providing an external assessment of model generalizability across taxonomic contrasts and demographic strata.
(CSV)

S1 Fig Impact of patient community state type on Voronoi-based decision boundary performance in BV prediction. (A) shows distribution of CSTs for each population group. (B-F) show tukey boxplot for boundary performances by CST for each population group. Boxplots are colored by the CST. The whiskers denote 1.5x IQR above the 75^th^ quartile and below the 25^th^ quartile. If this value is below the minimum or above the maximum, the whiskers represent those values instead.
(TIF)
